# Lack of association between polymorphisms in the CYP1A2 gene and risk of cancer: evidence from meta-analyses

**DOI:** 10.1186/s12885-016-2096-5

**Published:** 2016-02-10

**Authors:** Vladimir Vukovic, Carolina Ianuale, Emanuele Leoncini, Roberta Pastorino, Maria Rosaria Gualano, Rosarita Amore, Stefania Boccia

**Affiliations:** Institute of Public Health- Section of Hygiene, Università Cattolica del Sacro Cuore, Largo F.Vito 1, 00168 Rome, Italy

**Keywords:** CYP1A2, Polymorphism, Cancer, Meta-analysis, Susceptibility

## Abstract

**Background:**

Polymorphisms in the *CYP1A2* genes have the potential to affect the individual capacity to convert pre-carcinogens into carcinogens. With these comprehensive meta-analyses, we aimed to provide a quantitative assessment of the association between the published genetic association studies on *CYP1A2* single nucleotide polymorphisms (SNPs) and the risk of cancer.

**Methods:**

We searched MEDLINE, ISI Web of Science and SCOPUS bibliographic online databases and databases of genome-wide association studies (GWAS). After data extraction, we calculated Odds Ratios (ORs) and 95 % confidence intervals (CIs) for the association between the retrieved *CYP1A2* SNPs and cancer. Random effect model was used to calculate the pooled ORs. Begg and Egger tests, one-way sensitivity analysis were performed, when appropriate. We conducted stratified analyses by study design, sample size, ethnicity and tumour site.

**Results:**

Seventy case-control studies and one GWA study detailing on six different SNPs were included. Among the 71 included studies, 42 were population-based case-control studies, 28 hospital-based case-control studies and one genome-wide association study, including total of 47,413 cancer cases and 58,546 controls. The meta-analysis of 62 studies on rs762551, reported an OR of 1.03 (95 % CI, 0.96–1.12) for overall cancer (*P* for heterogeneity < 0.01; *I*^*2*^ = 50.4 %). When stratifying for tumour site, an OR of 0.84 (95 % CI, 0.70–1.01; *P* for heterogeneity = 0.23, *I*^*2*^ = 28.5 %) was reported for bladder cancer for those homozygous mutant of rs762551. An OR of 0.79 (95 % CI, 0.65–0.95; *P* for heterogeneity = 0.09, *I*^*2*^ = 58.1 %) was obtained for the bladder cancer from the hospital-based studies and on Caucasians.

**Conclusions:**

This large meta-analysis suggests no significant effect of the investigated *CYP1A2* SNPs on cancer overall risk under various genetic models. However, when stratifying according to the tumour site, our results showed a borderline not significant OR of 0.84 (95 % CI, 0.70–1.01) for bladder cancer for those homozygous mutant of rs762551. Due to the limitations of our meta-analyses, the results should be interpreted with attention and need to be further confirmed by high-quality studies, for all the potential *CYP1A2* SNPs.

## Background

Cancer is a complex disease that develops as a result of the interactions between environmental factors and genetic inheritance. In 2012 there were 14.1 million new cancer cases and 8.2 million cancer deaths worldwide [1]. Endogenous or exogenous xenobiotics are activated or inactivated through two metabolic steps by phase I and phase II enzymes [2]. The majority of chemical carcinogens require activation to electrophilic reactive forms to produce DNA adducts and this is mainly catalyzed by phase I enzymes. Although there are some exceptions, phase II enzymes, in contrast, detoxify such intermediates through conjugative reactions. The consequent formation of reactive metabolites and their binding to DNA to give stable adducts are considered to be critical in the carcinogenic process. It might therefore be expected that individuals with increased activation or low detoxifying potential have a higher susceptibility for cancer [3].

Cytochrome P450 1A2 (CYP1A2) enzyme is a member of the cytochrome P450 oxidase system and is involved in the phase I metabolism of xenobiotics. In humans, the CYP1A2 enzyme is encoded by the *CYP1A2* gene [4]. In vivo, CYP1A2 activity exhibits a remarkable degree of interindividual variations, as the gene expression is highly inducible by a number of dietary and environmental chemicals, including tobacco smoking, heterocyclic amines (HAs), coffee and cruciferous vegetables. Another possible contributor to interindividual variability in CYP1A2 activity is the occurrence of polymorphisms in the *CYP1A2* gene [5], which have the potential for determining individual’s different susceptibility to carcinogenesis [6]. CYP1A2 is expressed mainly in the liver, but also, expression of the CYP1A2 enzyme in pancreas and lung has been detected. The *CYP1A2* gene consists of 7 exons and is located at chromosome 15q22-qter. More than 40 single nucleotide polymorphisms (SNPs) of the *CYP1A2* gene have been discovered so far [7, 8].

High in vivo CYP1A2 activity has been suggested to be a susceptibility factor for cancers of the bladder, colon and rectum, where exposure to compounds such as aromatic amines and HAs has been implicated in the etiology of the disease [5, 6]. Additionally, it has been reported that among the *CYP1A2* polymorphisms, CYP1A2*1C (rs2069514) and CYP1A2*1 F (rs762551) are associated with reduced enzyme activity in smokers [5].

In recent years, efforts have been put into investigating the association of *CYP1A2* polymorphisms and the risk of several cancers, among them, colorectal [9–23], lung [7, 24–32], breast [33–46], bladder [4, 47–52], and other in different population groups, with inconsistent results. Therefore, with these meta-analyses we aimed to provide a quantitative assessment of the association between all *CYP1A2* polymorphisms and risk of cancer at various sites.

## Methods

### Selection criteria

Identification of the studies was carried out through a search of MEDLINE, ISI Web of Science and SCOPUS databases up to February 15^th^, 2015, by two independent researchers (R.A. and V.V.). The following terms were used: [(Cytochrome P450 1A2) OR (CYP1A2)] AND (Cancer) AND (Humans [MeSH]), without any restriction on language. All eligible studies were retrieved, and their bibliographies were hand-searched to find additional eligible studies. We only included published studies with full-text articles available.

Also, detail search of several publically available databases of genome-wide association studies (GWAS) - GWAS Central, Genetic Associations and Mechanisms in Oncology (GAME-ON), the Human Genome Epidemiology (HuGE) Navigator, National Human Genome Research Institute (NHGRI GWAS Catalog), The database of Genotypes and Phenotypes (dbGaP), The GWASdb, VarySysDB Disease Edition (VaDE), The genome wide association database (GWAS DB), was carried out up to February 15^th^, 2015 for the association between *CYP1A2* and various cancers using the combinations of following terms: (Cytochrome P450 1A2) OR (CYP1A2) OR (Chromosome 15q24.1) AND (Cancer). Additional consultation of principal investigators (PI) of the retrieved GWAS was undertaken in order to obtain the primary data and include them in the analyses.

Studies were considered eligible if they were assessing the frequency of any *CYP1A2* gene polymorphism in relation to the number of cancer cases and controls, according to the three variant genotypes (wild-type homozygous (wtwt), heterozygous (wtmt) and homozygous mutant (mtmt)). Case-only and case series studies with no control population were excluded, as well as studies based only on phenotypic tests, reviews, meta-analysis and studies focused entirely on individuals younger than 16 years old. When the same sample was used in several publications, we only considered the most recent or complete study to be used in our meta-analyses. Meanwhile, for studies that investigated more types of cancer, we counted them as individual data only in a subgroup analysis by the tumour type, while when they reported different ethnicity or location within the same study, we considered them as a separate studies.

### Data extraction

Two investigators (C.I. and V.V.) independently extracted the data from each article using a structured sheet and entered them into the database. The following items were considered: rs number, first author, year and location of the study, tumour site, ethnicity, study design, number of cases and controls, number of heterozygous and homozygous individuals for the *CYP1A2* polymorphisms in the compared groups. We used widely accepted National Center for Biotechnology Information (NCBI) CYP classification [53] to determine which specific genotype should be considered as wtwt, wtmt and mtmt. We also ranked studies according to their sample size, where studies with minimum of 200 cases were classified as small and above 200 cases as large.

### Statistical analysis

The estimated Odds Ratios (ORs) and 95 % confidence interval (CI) for the association between each *CYP1A2* SNP and cancer were defined as follows:

- wtmt vs wtwt (OR_1_)
- mtmt vs wtwt (OR_2_).

According to the following algorithm on the criteria to identify the best genetic model [54] for each SNP:

- Recessive model (mtmt versus wt carriers): if OR_2_ ≠ 1 and OR_1_ = 1
- Dominant model (mt carriers versus wtwt): if OR_2_ = OR_1_ ≠ 1,

we used the dominant model of inheritance for rs2069514, rs2069526 and rs35694136 and recessive model for rs762551, rs2470890 and rs2472304 in the meta-analysis. Random effect model was used to calculate the pooled ORs, taking into account the possibility of between studies heterogeneity [55], that was evaluated by the χ^2^-based Q statistics and the *I*^*2*^ statistics [56], where *I*^*2*^ = 0 % indicates no observed heterogeneity, within 25 % regarded as low, 50 % as moderate, and 75 % as high [57]. A visual inspection of Begg’s funnel plot and Begg’s and Egger’s asymmetry tests [58] were used to investigate publication bias, where appropriate [59]. To determinate the deviation from the Hardy-Weinberg Equilibrium (HWE) we used a publicly available program (http://ihg.gsf.de/cgi-bin/hw/hwa1.pl ). Additionally, the Galbraith’s test [60] was performed to evaluate the weight each study had on the overall estimate and its contribution on Q-statistics. We also performed a one-way sensitivity analysis to explore the effect that each study had on the overall effect estimate, by computing the meta-analysis estimates repeatedly after every study has been omitted.

Studies whose allele frequency in the control population deviated significantly from the Hardy-Weinberg Equilibrium (HWE) at the *p*-value ≤ 0.01 were excluded from the meta-analyses, given that this deviation may represent bias. We conducted stratified analysis by study design, ethnicity, sample size and tumour site to investigate the potential sources of heterogeneity across the studies. Statistical analyses were performed using the STATA software package v. 13 (Stata Corporation, College 162 Station, TX, USA), and all statistical tests were two-sided.

## Results

### Characteristics of the studies

We identified a total of 2541 studies through MEDLINE, ISI Web of Science and SCOPUS online databases. One thousand and sixteen studies were left after duplicates removal, and after carefully reading the titles, only 175 studies were assessed for eligibility. After reviewing the abstracts, 120 full text articles were obtained for further eligibility. By not fulfilling the inclusion criteria, 61 full text articles were excluded, leaving 59 studies for quantitative synthesis. Additional hand-search of the reference lists of 59 included studies was done and 11 new eligible studies were found, resulting in 70 included studies.

**Fig. 1:**
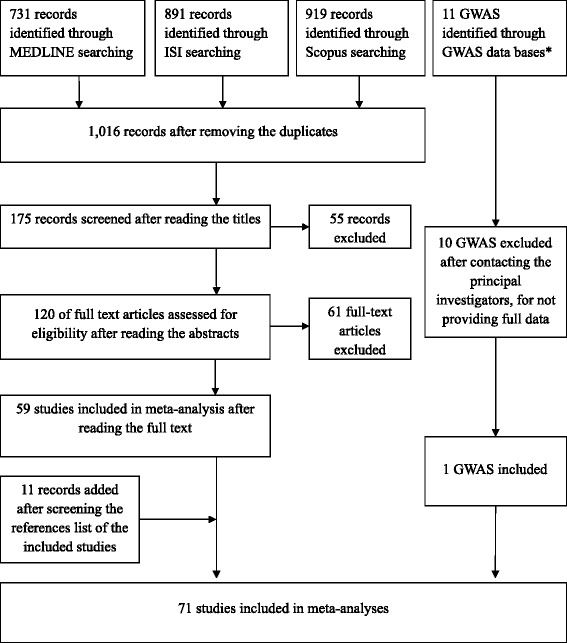
Flowchart depicting literature search and study selection. *GWAS data bases searched: GWAS Central, Genetic Associations and Mechanisms in Oncology (GAME-ON), the Human Genome Epidemiology (HuGE) Navigator, National Human Genome Research Institute (NHGRI GWAS Catalog), The database of Genotypes and Phenotypes (dbGaP), The GWASdb, VarySysDB Disease Edition (VaDE), The genome wide association database (GWAS DB)

Eleven GWASs on the association between *CYP1A2* SNPs and cancer risk were identified after detail search of GWAS online databases. Studies did not report full data on investigated SNPs, so we contacted principal investigators (PIs) to retrieve the information and include into our analyses. After 3 repeated solicitations, only one PI provided us with the full data on *CYP1A2* SNPs of breast cancer cases and controls, and by this making total of 71 studies included in our meta-analyses [4, 7–52, 61–84]. Figure 1 shows the process of literature search and study selection.

Among the 71 included studies, 42 were population-based case-control studies, 28 hospital-based case-control studies and one genome-wide association study, including total of 47,413 cancer cases and 58,546 controls (Table 1). The total investigated SNPs were six, of which 62 studies on the rs762551 [4, 7–21, 23, 24, 26–46, 48–50, 52, 61–65, 67, 68, 72–75, 77–79, 81–84]. Thirty five studies out of 62 were conducted on Caucasians (56.5 %), 17 on mixed populations (27.4 %) and 10 on Asians (16.1 %), including 33,181 cancer cases and 40,195 controls. Among them, 15 were on breast cancer, 14 studies on colorectal, and 9 on lung cancer.

**Table 1:** Description of 45 studies included in meta-analysis of association between different *CYP1A2* SNPs and cancer

Rs number	First author	Year	Tumour site	Country	Ethnicity	Sample size (No. cases/controls)	Crude OR° (95 % CI) recessive model	Crude OR (95 % CI) dominant model
rs762551	Goodman MT. [73]	2001	Ovaries	USA	Mixed	116/138*^a^	0.52 (0.19–1.43)	–
	Sachse C. [18]	2002	Colorectum	UK	Caucasian	490/593*ª	1.15 (0.70–1.88)	–
	Goodman MT. [74]	2003	Ovaries	USA	Mixed	164/194*ª	0.73 (0.34–1.55)	–
	Hopper J. [36]	2003	Breast	Australia	Caucasian	204/287*^c^	0.55 (0.27–1.13)	–
	Doherty JA. [68]	2005	Endometrium	USA	Mixed	371/420*ª	1.27 (0.75–2.15)	–
	Landi S. [16]	2005	Colorectum	Spain	Caucasian	361/321*^b^	**1.74 (1.05–2.88)**	–
	Le Marchand L. [39]	2005	Breast	USA	Mixed	1339/1369*^a^	**0.73 (0.55–0.96)**	–
	Prawan A. [81]	2005	Liver	Thailand	Asian	216/233*^a^	0.52 (0.24–1.13)	–
	Mochizuki J. [79]	2005	Liver	Japan	Asian	31/123*^a^	1.35 (0.26–7.01)	–
	Agudo A. [61]	2006	Stomach	European countries^1^	Caucasian	242/943*^a^	0.88 (0.50–1.55)	–
	Bae SY. [9]	2006	Colorectum	S. Korea	Asian	111/93*^b^	1.14 (0.51–2.54)	–
	De Roos AJ. [67]	2006	Lymphoma	USA	Mixed	745/640*^a^	0.91 (0.63–1.31)	–
	Li D. [8]	2006	Pancreas	USA	Mixed	307/333*^b^	1.10 (0.65–1.84)	–
	Long JR. [41]	2006	Breast	China	Asian	1082/1139*^a^	0.89 (0.71–1.13)	–
	Rebbeck TR [82]	2006	Endometrium	USA	Mixed	475/1233*^a^	1.03 (0.73–1.46)	–
	Kiss I. [13]	2007	Colorectum	Hungary	Caucasian	500/500*^b^	1.07 (0.74–1.54)	–
	Kury S. [15]	2007	Colorectum	France	Caucasian	1013/1118*^a^	1.03 (0.75–1.41)	–
	Osawa Y. [29]	2007	Lung	Japan	Asian	103/111*^a^	1.17 (0.57–2.42)	–
	Takata Y. [46]	2007	Breast	USA (Hawaii)	Mixed	325/250*^a^	0.76 (0.39–1.49)	–
	Yoshida K. [23]	2007	Colorectum	Japan	Asian	64/111*^a^	0.57 (0.21–1.53)	–
	Gemignani F. [26]	2007	Lung	European countries^2^	Caucasian	297/310*^b^	0.86 (0.50–1.49)	–
	Kotsopoulos J. [38]	2007	Breast	Canada	Caucasian	170/241*^b^	**2.12 (0.99–4.57)**	–
	Gulyaeva LF. [35]	2008	Endometrium	Russia	Caucasian	166/180*^a^	2.20 (0.40–12.16)	–
	Gulyaeva LF. [35]	2008	Ovaries	Russia	Caucasian	96/180*^a^	**9.21 (1.95–43.53)**	–
	Gulyaeva LF. [35]	2008	Breast	Russia	Caucasian	93/180*^a^	**27.58 (6.32–120.35)**	–
	Hirata H. [75]	2008	Endometrium	USA	Caucasian	150/165*^a^	0.96 (0.62–1.51)	–
	Saebo M. [19]	2008	Colorectum	Norway	Caucasian	198/222*^a^	1.05 (0.49–2.23)	–
	Suzuki H. [84]	2008	Pancreas	USA	Caucasian	649/585*^a^	0.93 (0.56–1.54)	–
	Figueroa JD [48]	2008	Bladder	Spain	Caucasian	1101/1021*^b^	0.80 (0.62–1.04)	–
	Zienolddiny S. [32]	2008	Lung	Norway	Caucasian	335/393*^a^	1.43 (0.88–2.32)	–
	Cotterchio M. [11]	2008	Colorectum	Canada	Caucasian	835/1247*^a^	0.91 (0.67–1.23)	–
	Aldrich MC. [7]	2009	Lung	USA	Mixed	113/299*^a^	**3.36 (1.58–7.13)**	–
	Altayli E. [4]	2009	Bladder	Turkey	Caucasian	135/128*^b^	1.51 (0.88–2.60)	–
	B’chir F. [24]	2009	Lung	Tunisia	Caucasian	101/98*^b^	0.90 (0.47–1.70)	–
	Kobayashi M. [78]	2009	Stomach	Japan	Asian	141/286*^b^	0.62 (0.33–1.18)	–
	Kobayashi M. [14]	2009	Colorectum	Japan	Asian	104/225*^b^	0.64 (0.31–1.32)	–
	Shimada N (a) [45]	2009	Breast	Japan and Brazil	Asian	483/484*^b^	1.02 (0.71–1.47)	–
	Shimada N (b) [45]	2009	Breast	Brazil	Mixed	389/389*^b^	**0.50 (0.31–0.80)**	–
	Sangrajrang S. [44]	2009	Breast	Thailand	Asian	552/483*^b^	**2.72 (1.52–4.86)**	–
	Villanueva C. [52]	2009	Bladder	Spain	Caucasian	1034/911*^b^	0.82 (0.62–1.07)	–
	Canova C. [64]	2009	UADT	European countries^3^	Caucasian	1480/1437*^b^	0.88 (0.69–1.13)	–
	Cleary SP [10]	2010	Colorectum	Canada	Caucasian	1165/1290*^a^	0.93 (0.71–1.22)	–
	Pavanello S. [50]	2010	Bladder	Italy	Caucasian	155/161*^b^	0.57 (0.25–1.30)	–
	Singh A. [31]	2010	Lung	India	Caucasian	200/200*^a^	**0.61 (0.37–1.00)**	–
	The MARIE-GENICA Consortium [43]	2010	Breast	Germany	Caucasian	3147/5485*^a^	1.04 (0.88–1.22)	–
	Canova C. [65]	2010	UADT	Italy	Caucasian	376/386*^b^	1.21 (0.77–1.89)	–
	Ashton KA [62]	2010	Endometrium	Australia	Caucasian	191/291*^a^	1.03 (0.71–1.49)	–
	Guey LT [49]	2010	Bladder	Spain	Caucasian	1005/1021*^b^	**0.77 (0.58–1.00)**	–
	Rudolph A. [17]	2011	Colorectum	Germany	Caucasian	678/680*^a^	1.38 (0.93–2.05)	–
	Sainz J. [20]	2011	Colorectum	Germany	Caucasian	1764/1786*^a^	0.95 (0.75–1.19)	–
	Jang JH [77]	2012	Pancreas	Canada	Mixed	447/880*^a^	1.08 (0.73–1.59)	–
	Khvostova EP [37]	2012	Breast	Russia	Caucasian	323/526*^b^	**1.82 (1.14–2.90)**	–
	Pavanello S. [30]	2012	Lung	Denmark	Caucasian	421/776*^a^	**1.63 (1.08–2.48)**	–
	Wang J. [21]	2012	Colorectum	USA	Mixed	305/357*^a^	0.97 (0.55–1.70)	–
	Anderson LN [33]	2012	Breast	Canada	Mixed	886/932*^a^	**1.50 (1.09–2.07)**	–
	Ayari I. [34]	2013	Breast	Tunisia	Caucasian	117/42*^b^	1.62 (0.51–5.11)	–
	Barbieri RB [63]	2013	Thyroid gland	Brasil	Mixed	123/339*^a^	**2.12 (1.16–3.87)**	–
	Dik VK [12]	2013	Colorectum	The Netherlands	Caucasian	970/1590*^a^	1.10 (0.85–1.43)	–
	Gervasini G. [27]	2013	Lung	Spain	Caucasian	95/196*^b^	1.25 (0.60–2.61)	–
	Lee HJ. [40]	2013	Breast	USA	Mixed	579/981*^a^	1.22 (0.85–1.75)	–
	Lowcock E. [42]	2013	Breast	Canada	Mixed	1693/1761*^a^	1.24 (0.97–1.57)	–
	Ghoshal U. [72]	2014	Stomach	India	Caucasian	88/170*^a^	1.13 (0.57–2.22)	–
	Mikhalenko AP. [28]	2014	Lung	Belarus	Caucasian	92/328*^a^	1.14 (0.44–2.93)	–
	Shahabi A. [83]	2014	Prostate	USA	Mixed	1480/777*^a^	0.97 (0.72–1.30)	–
rs2069514	Sachse C. [18]	2002	Colorectum	UK	Caucasian	60/73*^a^	–	**12.71 (1.56–103.44)**
	Tsukino H. [51]	2004	Bladder	Japan	Asian	306/306*^a^	–	0.95 (0.69–1.31)
	Landi S. [16]	2005	Colorectum	Spain	Caucasian	328/295*^b^	–	0.90 (0.38–2.10)
	Chiou HL [25]	2005	Lung	China	Asian	162/208*^b^	–	1.04 (0.69–1.57)
	Agudo A. [61]	2006	Stomach	European countries^1^	Caucasian	243/945*^a^	–	1.66 (0.72–3.84)
	Chen X. [66]	2006	Liver	China	Asian	430/546*^a^	–	0.97 (0.75–1.24)
	Bae SY. [9]	2006	Colorectum	S. Korea	Asian	111/93*^b^	–	0.68 (0.39–1.18)
	Yoshida K. [23]	2007	Colorectum	Japan	Asian	66/113*^a^	–	0.82 (0.44–1.52)
	Osawa Y. [29]	2007	Lung	Japan	Asian	106/113*^a^	–	0.80 (0.46–1.36)
	Gemignani F. [26]	2007	Lung	European countries^2^	Caucasian	278/294*^b^	–	0.52 (0.16–1.75)
	Zienolddiny S. [32]	2008	Lung	Norway	Caucasian	243/214*^a^	–	0.65 (0.22–1.91)
	Imaizumi T. [76]	2009	Liver	Japan	Asian	209/256*^a^	–	0.88 (0.61–1.27)
	B’chir F. [24]	2009	Lung	Tunisia	Caucasian	101/98*^b^	–	**5.88 (2.96–11.70)**
	Yeh CC [22]	2009	Colorectum	Taiwan	Asian	718/731*^b^	–	1.08 (0.88–1.32)
	Gemignani F. [71]	2009	Pleura	Italy	Caucasian	92/643*^b^	–	0.33 (0.04–2.45)
	Singh A. [31]	2010	Lung	India	Caucasian	200/200*^a^	–	0.84 (0.47–1.50)
	Pavanello S. [30]	2012	Lung	Denmark	Caucasian	423/777*^a^	–	0.85 (0.32–2.24)
	Ayari I. [34]	2013	Breast	Tunisia	Caucasian	109/41*^b^	–	**0.35 (0.14–0.90)**
	Gervasini G. [27]	2013	Lung	Spain	Caucasian	95/196*^b^	–	2.67 (0.70–10.17)
	Cui X. [47]	2013	Bladder	Japan	Asian	282/257*^b^	–	0.89 (0.63–1.26)
rs2069526	Sachse C. [18]	2002	Colorectum	UK	Caucasian	490/593*^a^	–	0.86 (0.60–1.22)
	Landi S. [16]	2005	Colorectum	Spain	Caucasian	321/288*^b^	–	1.27 (0.55–2.90)
	Gemignani F. [26]	2007	Lung	European countries^2^	Caucasian	247/251*^b^	–	**0.34 (0.14–0.81)**
	Zienolddiny S. [32]	2008	Lung	Norway	Caucasian	194/239*^a^	–	1.66 (0.37–7.49)
	Gemignani F. [71]	2009	Pleura	Italy	Caucasian	78/579*^b^	–	1.10 (0.42–2.90)
	Singh A. [31]	2010	Lung	India	Caucasian	200/200*^a^	–	1.07 (0.65–1.75)
	Gervasini G. [27]	2013	Lung	Spain	Caucasian	95/196*^b^	–	1.36 (0.57–3.27)
rs2470890	Hopper J. [36]	2003	Breast	Australia	Caucasian	204/287*^c^	0.82 (0.47–1.43)	–
	Landi S. [16]	2005	Colorectum	Spain	Caucasian	353/320*^b^	1.24 (0.84–1.82)	–
	Chen X. [66]	2006	Liver	China	Asian	428/545*^a^	0.53 (0.27–1.06)	–
	Kury S. [15]	2007	Colorectum	France	Caucasian	1013/1118*^a^	1.07 (0.90–1.27)	–
	Gemignani F. [26]	2007	Lung	European countries^2^	Caucasian	283/298*^b^	0.83 (0.51–1.35)	–
	Aldrich MC. [7]	2009	Lung	USA	Mixed	113/299*^a^	1.12 (0.59–2.13)	–
	Gemignani F. [71]	2009	Pleura	Italy	Caucasian	85/669*^b^	1.02 (0.56–1.88)	–
	Canova C. [64]	2009	UADT	European countries^3^	Caucasian	1455/1403*^b^	1.03 (0.84–1.26)	–
	Canova C. [65]	2010	UADT	Italy	Caucasian	374/387*^b^	**1.51 (1.02–2.23)**	–
	Anderson LN [33]	2012	Breast	Canada	Mixed	884/927*^a^	**1.49 (1.18–1.89)**	–
	Eom SY. [69]	2013	Stomach	S. Korea	Asian	473/472*^b^	1.15 (0.55–2.37)	–
rs2472304	Hopper J. [36]	2003	Breast	Australia	Caucasian	204/286*^c^	0.81 (0.46–1.43)	–
	Sangrajrang S. [44]	2009	Breast	Thailand	Asian	552/478*^b^	1.16 (0.59–2.29)	–
	Aldrich MC. [7]	2009	Lung	USA	Mixed	112/297*^a^	1.12 (0.59–2.14)	–
	Ferlin A. [70]	2010	Testicles	Italy	Caucasian	234/218*^a^	**0.68 (0.46–1.01)**	–
rs35694136	Li D. [8]	2006	Pancreas	USA	Mixed	307/329*^b^	–	0.87 (0.63–1.18)
	Olivieri EH [80]	2009	Head and Neck	Brasil	Mixed	81/134*^b^	–	**8.98 (4.49–17.93)**
	Pavanello S. [50]	2010	Bladder	Italy	Caucasian	167/141*^b^	–	0.73 (0.46–1.14)
	Singh A. [31]	2010	Lung	India	Caucasian	200/200*^a^	–	**1.65 (1.11–2.45)**
	Pavanello S. [30]	2012	Lung	Denmark	Caucasian	415/760*^a^	–	0.98 (0.65–1.49)
	Ayari I. [34]	2013	Breast	Tunisia	Caucasian	108/38*^b^	–	0.88 (0.40–1.93)

Statistically significant results are presented in bold. °OR (95 % CI) Odds Ratio and 95 % Confidence Interval ^1^Ten European countries: Denmark, France, Germany, Greece, Italy, the Netherlands, Norway, Spain, Sweden, and the United Kingdom. ^2^Six European countries: Romania, Hungary, Poland, Russia, Slovakia, Czech Republic. ^3^Ten European countries: Czech Republic, Germany, Greece, Italy, Ireland, Norway, United Kingdom, Spain, Croatia, France. *Hardy-Weinberg Equilibrium (HWE), *P* value ˃0.01. ^a^Population-based study ^b^Hospital-based study ^c^Genome-wide Association Study. (a), (b) One study with two different population

Twenty studies investigated the rs2069514 [9, 16, 18, 22–27, 29–32, 34, 47, 51, 61, 66, 71, 76], of which 11 were conducted on Caucasians (55 %) and 9 on Asians (45 %). Eight studies investigated the effect on lung cancer (40 %), 5 studies on colorectal cancer (25 %), 2 on liver cancer (10 %), 2 on bladder (10 %) and by 1 study on stomach (5 %), breast (5 %) and pleura (5 %), totaling for 4562 cancer cases and 6399 controls (Table 1).

The remaining four SNPs were investigated by a reduced number of studies and details are presented in Table 1. Genotype frequencies in all control groups did not deviate from values predicted by HWE (Table 1). As some studies on different cancer types shared the same control group [35], these studies were aggregated when performing the meta-analyses, except when stratified by tumour site.

### Quantitative synthesis

As the crude analysis for rs762551 provided an OR_1_ of 1.03 (95 % CI 0.98–1.07) and an OR_2_ of 1.06 (95 % CI 0.97–1.16), for rs2470890 OR_1_ 1.03 (95 % CI 0.93–1.14) and OR_2_ of 1.14 (95 % CI 0.97–1.34) and for rs2472304 OR_1_ of 0.98 (95 % CI 0.79–1.22) and OR_2_ of 0.89 (95 % CI 0.66–1.22) according to the criteria proposed in the methods section, we applied the recessive model of inheritance for the meta-analyses. On the other hand, for rs2069514, rs2069526 and rs35694136 original papers did not report enough data to calculate OR1 and OR2, so we were able only to apply the dominant model for the data analyses.

**Fig. 2:**
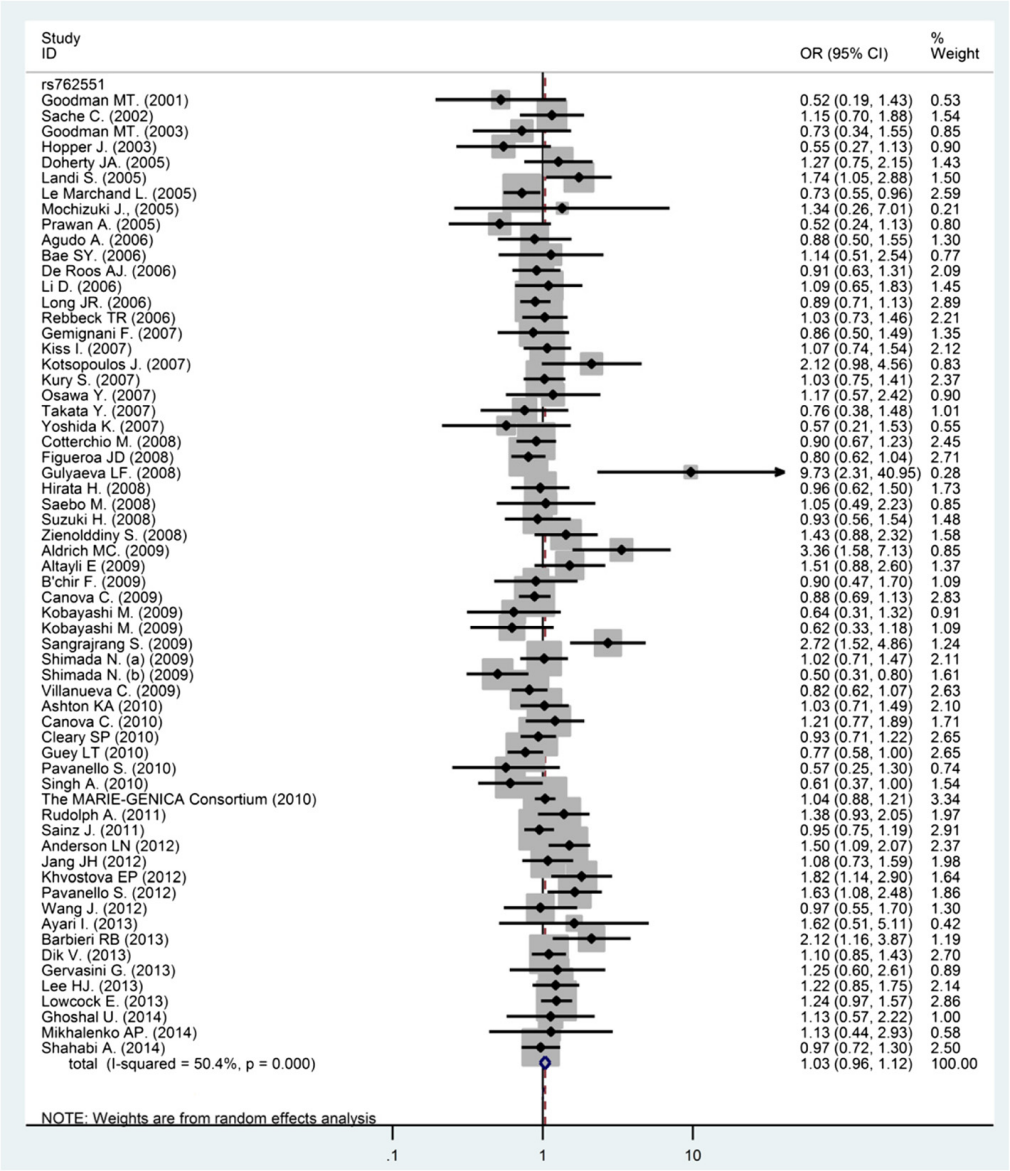
Forest plot of the *CYP1A2* rs762551 and cancer meta-analysis under recessive models of inheritance. The diamonds and horizontal lines correspond to the study-specific odds ratio (OR) and 95 % confidence interval (CI)

**Fig. 3:**
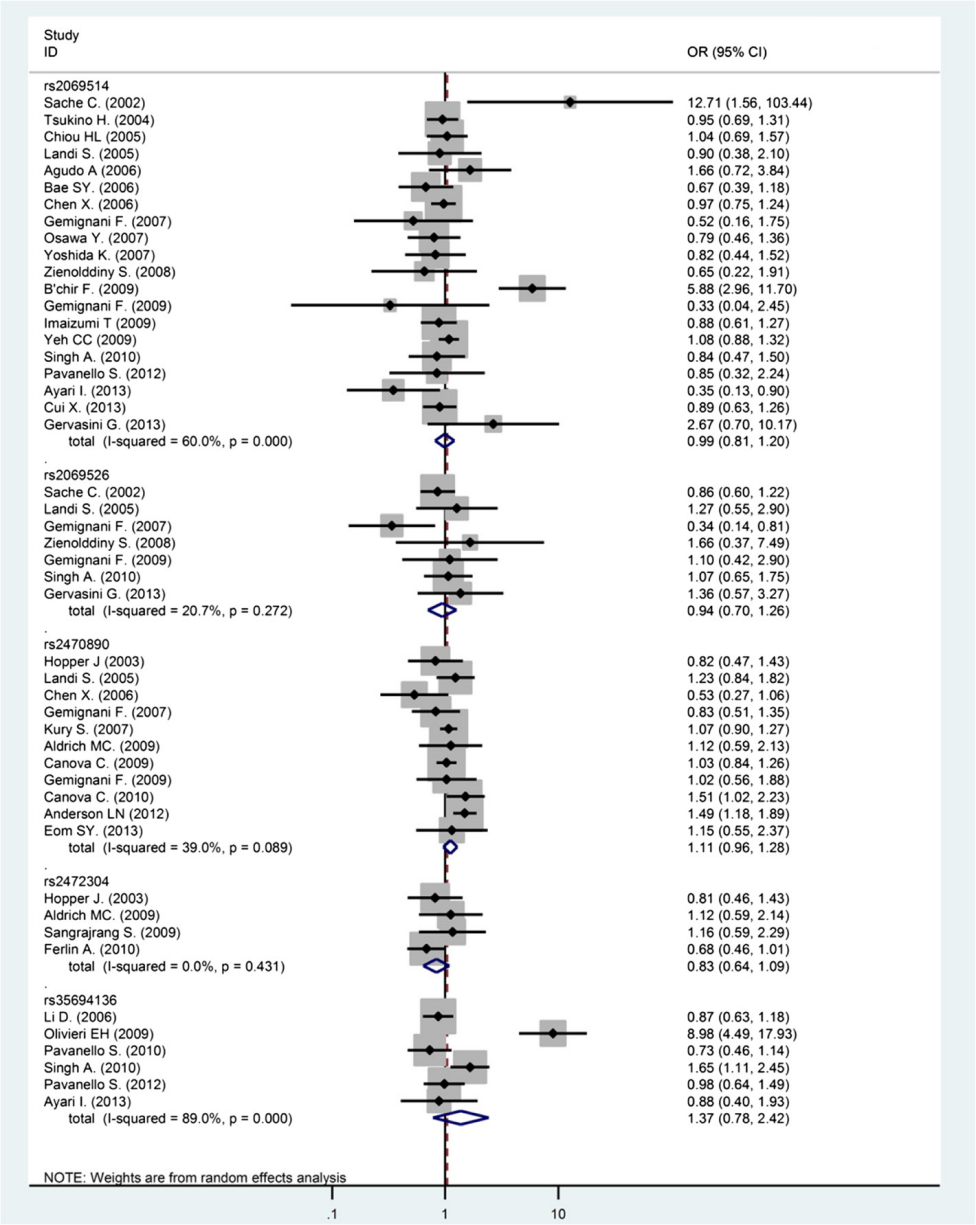
Forest plot of the remaining five *CYP1A2* SNPs and cancer meta-analyses under different models of inheritance. The diamonds and horizontal lines correspond to the study-specific odds ratio (OR) and 95 % confidence interval (CI)

**Fig. 4:**
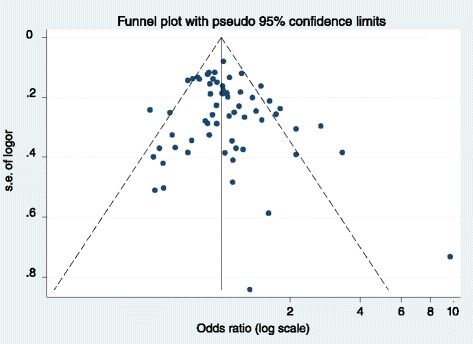
Funnel plot for publication bias for studies with *CYP1A2* rs762551. Each point represents an individual study for the indicated association

The Figs. 2 and 3 depict the forest plots of the ORs of the six *CYP1A2* SNPs and cancer. By pooling 62 studies on rs762551, the meta-analysis reported an OR of 1.03 (95 % CI 0.96–1.12) for overall cancer (*P* for heterogeneity < 0.01; *I*^*2*^ = 50.4 %). Egger test and the Begg’s correlation method did not provide statistical evidence of publication bias (*P* = 0.19 and *P* = 0.39, respectively) (Fig. 4). To explore the potential sources of heterogeneity, we performed the Galbraith’s test which identified the study of Shimada N. (b) [45] and Sangrajrang S. [44], as the main contributors to heterogeneity (graph not shown). In the one-way sensitivity analysis, these two outlying studies were omitted from meta-analysis and the overall OR slightly changed to 1.03 (95 % CI 0.96–1.11), with a reduced heterogeneity (*P* for heterogeneity <0.01; *I*^*2*^ = 43.0 %).

Results of the stratified meta-analyses are reported in the Table 2. When stratifying the results of meta-analysis for rs762551 by ethnicity, we found no significant effect of *CYP1A2* on cancer risk for Caucasians (OR = 1.03; 95 % CI 0.94–1.13), Asians (OR = 0.95; 95 % CI 0.72–1.27) nor among a mixed population (OR = 1.05; 95 % CI 0.89–1.25). When stratifying according to the tumour site, results showed an OR of 0.84 (95 % CI 0.70–1.01; *P* for heterogeneity = 0.23, *I*^*2*^ = 28.5 %) for bladder cancer for those homozygous mutant types of rs762551 (Table 2). We further examined the association between the *CYP1A2* polymorphism and cancer risk according to ethnicity, source of controls and sample size and then stratified by cancer type. We found a significant OR of 0.79 (95 % CI 0.65–0.95; *P* for heterogeneity = 0.09, *I*^*2*^ = 58.1 %) for bladder cancer among the hospital-based population and among Caucasians. There was no significant association among Caucasians for breast cancer (OR = 1.71; 95 % CI 0.94–3.10; *P* for heterogeneity < 0.01, *I*^*2*^ = 83.4 %), lung cancer (OR = 1.07; 95 % CI 0.79–1.44; *P* for heterogeneity = 0.07, *I*^*2*^ = 48.1 %,) or colorectal cancer (OR = 1.05, 95 % CI 0.94–1.16; *P* for heterogeneity = 0.49, *I*^*2*^ = 0.0 %). Among Asians, when stratifying for cancer type, we obtained an OR of 0.76 (95 % CI 0.47–1.22; *P* for heterogeneity = 0.48, *I*^*2*^ = 0.0 %) for colorectal cancer and OR = 1.27 (95 % CI 0.75–2.16; *P* for heterogeneity <0.01, *I*^*2*^ = 83.6 %) for breast cancer.

**Table 2:** Subgroup meta-analyses of *CYP1A2* SNPs and cancer risk according to study design, ethnicity and tumour site

	Number cases/controls		Recessive model			
	Exposed	Not exposed	OR°	95 % CI°	*I* ^*2*^ (%)	*P* value for heterogeneity
rs762551	3373/4006	29,808/36,562	1.03	0.96–1.12	50.4	<0.01
Study design						
Hospital based	1048/1110	8289/8482	1.03	0.88–1.20	60.3	<0.01
Population based	2314/2869	21,326/27,820	1.05	0.96–1.15	41.8	<0.01
Study sample size						
Large	2883/3387	27,381/32,680	1.02	0.94–1.11	55.9	<0.01
Small	490/619	2427/3882	1.09	0.90–1.32	36.0	0.05
Ethnicity						
Asian	348/414	2539/2874	0.95	0.72–1.27	54.6	0.02
Caucasian	2132/2600	18,305/23,388	1.03	0.94–1.13	42.4	<0.01
Mixed	893/992	8964/10,300	1.05	0.89–1.25	62.8	<0.01
Tumour site						
Bladder	392/436	3038/2806	**0.84**	**0.70–1.01**	28.5	0.23
Breast	1097/1280	10,285/13,269	1.17	0.94–1.45	79.2	<0.01
Colorectum	803/934	7755/9199	1.03	0.93–1.14	0.0	0.56
Endometrium	258/391	1095/1898	1.06	0.87–1.30	0.0	0.85
Liver	12/26	235/330	0.63	0.30–1.32	5.0	0.31
Lung	221/265	1536/2446	1.20	0.87–1.64	58.9	0.01
Ovaries	27/34	349/478	1.31	0.33–5.19	80.3	0.01
Pancreas	107/142	1296/1656	1.04	0.80–1.36	0.0	0.87
Stomach	46/141	425/1258	0.85	0.59–1.21	0.0	0.45
UADT	186/192	1670/1631	0.97	0.73–1.29	29.9	0.23
	Number cases/controls		Dominant model			
	Exposed	Not exposed	OR	95 % CI	*I* ^*2*^ (%)	*P* value for heterogeneity
rs2069514	1229/1373	3333/5026	0.99	0.81–1.21	60.0	<0.01
Study design						
Hospital based	758/783	1727/2329	1.01	0.73–1.40	73.7	<0.01
Population based	471/590	1606/2697	0.94	0.78–1.14	10.6	0.35
Study sample size						
Large	969/1085	2691/3736	0.97	0.86–1.09	0.0	0.89
Small	260/288	642/1290	1.18	0.65–2.11	81.1	<0.01
Ethnicity						
Asian	1093/1235	1297/1388	0.96	0.86–1.07	0.0	0.86
Caucasian	136/138	2036/3638	1.16	0.63–2.14	75.7	<0.01
Tumour site						
Bladder	236/237	352/326	0.92	0.73–1.17	0.0	0.81
Colorectum	447/458	836/847	0.96	0.65–1.43	53.2	0.07
Liver	315/409	324/393	0.94	0.76–1.15	0.0	0.68
Lung	211/219	1397/1881	1.16	0.68–1.99	76.3	<0.01
	Number cases/controls		Dominant model			
	Exposed	Not exposed	OR	95 % CI	*I* ^*2*^ (%)	*P* value for heterogeneity
rs2069526	139/202	1486/2144	0.94	0.70–1.26	20.7	0.27
Study design						
Hospital based	35/78	706/1236	0.89	0.47–1.72	53.9	0.09
Population based	104/124	780/908	0.94	0.71–1.25	0.0	0.59
Study sample size						
Large	121/151	1137/1181	0.85	0.56–1.28	49.4	0.12
Small	18/51	349/963	1.29	0.71–2.35	0.0	0.89
Ethnicity						
Caucasian	139/202	1486/2144	0.94	0.70–1.26	20.7	0.27
Tumour site						
Colorectum	74/93	737/788	0.91	0.66–1.26	0.0	0.40
Lung	60/75	676/811	0.90	0.47–1.71	55.4	0.08
	Number cases/controls		Recessive model			
	Exposed	Not exposed	OR	95 % CI	*I* ^*2*^ (%)	*P* value for heterogeneity
rs2470890	1106/1187	4559/5538	1.11	0.96–1.28	39.0	0.09
Study design						
Hospital based	429/480	2594/3069	1.10	0.95–1.27	0.0	0.46
Population based	655/670	1783/2219	1.09	0.80–1.50	70.5	0.02
Study sample size						
Large	1077/1043	4390/4714	1.11	0.94–1.30	50.9	0.04
Small	29/144	169/824	1.07	0.69–1.66	0.0	0.85
Ethnicity						
Asian	28/42	873/975	0.77	0.37–1.64	55.4	0.13
Caucasian	863/957	2904/3525	1.07	0.96–1.20	0.0	0.47
Mixed	215/188	782/1038	**1.44**	**1.16–1.80**	0.0	0.41
Tumour site						
Breast	222/189	866/1025	1.17	0.65–2.08	73.4	0.05
Colorectum	500/509	866/929	1.10	0.94–1.28	0.0	0.51
Lung	48/77	348/520	0.92	0.63–1.37	0.0	0.47
UADT	294/262	1535/1528	1.20	0.83–1.74	65.7	0.09
	Number cases/controls		Recessive model			
	Exposed	Not exposed	OR	95 % CI	*I* ^*2*^ (%)	*P* value for heterogeneity
rs2472304	127/172	975/1107	0.84	0.64–1.09	0.0	0.43
Study design						
Population based	85/120	261/395	0.82	0.51–1.30	40.4	0.20
Study sample size						
Large	112/136	878/846	0.79	0.59–1.05	0.0	0.41
Ethnicity						
Caucasian	92/121	346/383	**0.72**	**0.52–0.99**	0.0	0.61
Tumour site						
Breast	42/52	714/712	0.94	0.61–1.45	0.0	0.43
	Number cases/controls		Dominant model			
	Exposed	Not exposed	OR	95 % CI	*I* ^*2*^ (%)	*P* value for heterogeneity
rs35694136	439/419	839/1183	1.37	0.78–2.42	89.0	<0.01
Study design						
Hospital based	290/263	373/379	1.46	0.56–3.77	92.7	<0.01
Population based	149/156	466/804	1.28	0.77–2.13	68.4	0.08
Study sample size						
Large	290/319	632/970	1.11	0.75–1.64	69.8	0.04
Small	149/100	207/213	1.78	0.37–8.60	94.6	<0.01
Ethnicity						
Caucasian	255/241	635/898	1.04	0.70–1.53	62.0	0.05
Mixed	184/178	204/285	2.73	0.28–27.09	97.3	<0.01
Tumour site						
Lung	149/156	466/804	1.28	0.77–2.13	68.4	0.08

Statistically significant ORs are presented in bold. °OR (95 % CI) Odds Ratio and 95 % Confidence Interval

When pooling the 20 studies on rs2069514, the meta-analysis provided an OR of 0.99 (95 % CI 0.81–1.21) for overall cancer (*P* for heterogeneity <0.01; *I*^*2*^ = 60 %) (Fig. 2). Egger test and the Begg’s correlation method provided no statistical evidence of publication bias (*P* = 0.86 and *P* = 0.56, respectively). We performed the Galbraith’s test to explore the source of heterogeneity and accordingly singled out the study of B’chir F. et al. [24] as the main contributor to heterogeneity (graph not shown). In the one-way sensitivity analysis, the study of B’chir F. et al. [24] was omitted from the overall meta-analysis and the heterogeneity dropped down to 14 % (*P* = 0.28), with the OR of 0.93 (95 % CI 0.82–1.06).

We evaluated the effect of the rs2069514 polymorphism according to the tumour site and obtained an OR of 0.96 (95 % CI 0.65–1.43; *P* for heterogeneity = 0.07, *I*^*2*^ = 53.2 %) for colorectal cancer, an OR of 1.29 (95 % CI 0.60–2.79; *P* for heterogeneity = 0.00; *I*^*2*^ = 82.1 %) for lung cancer (Table 2). Analyses on different ethnicity and study design did not provide any significant results (Caucasians OR = 1.16; 95 % CI 0.63–2.14; *I*^*2*^ = 75.7 %, *P* < 0.01, for Asians OR = 0.96; 95 % CI 0.86–1.07, *I*^*2*^ = 0.0 %; *P* = 0.86 and Hospital-based study design OR = 1.01; 95 % CI 0.73–1.40; *I*^*2*^ = 73.7 %, *P* < 0.01, for Population-based design OR = 0.94; 95 % CI 0.78–1.14; *I*^*2*^ = 10.6 %, *P* = 0.35). We did not observe any significant association between rs2069514 polymorphism and cancer risk when subgrouping data according to ethnicity, source of controls and sample size and then stratified by cancer type. Among Caucasians, we obtained an OR of 1.28 (95 % CI 0.55–2.98; *I*^*2*^ = 80.9 %, *P* < 0.01) for lung cancer, while among Asians OR = 0.94 (95 % CI 0.68–1.31; *I*^*2*^ = 0.0 %, *P* = 0.44) for lung and OR = 0.94 (95 % CI 0.71–1.24; *I*^*2*^ = 28.8 %, *P* = 0.25) for colorectal cancer.

We performed meta-analysis of 11 studies on rs2470890 which provided an OR of 1.11 (95 % CI 0.96-1.28) for the overall cancer risk (*P* for heterogeneity 0.09; *I*^*2*^ = 39 %) (Fig. 2). Egger test and the Begg’s correlation method provided no statistical evidence of publication bias (*P* = 0.42 and *P* = 0.59, respectively). The Galbraith’s test singled out the study of Anderson LN et al. [33] as the main contributor to heterogeneity (graph not shown). In one-way sensitivity analysis, this study was omitted from the overall meta-analysis and the heterogeneity dropped down to 6 % (*P* = 0.39), with still not significant OR of 1.06 (95 % CI, 0.94–1.19). The effect of rs2470890 polymorphism according to the tumour site was also evaluated and was obtained non-significant result of OR of 1.10 (95 % CI, 0.94–1.28) *P* for heterogeneity = 0.51, *I*^*2*^ = 0.0 % for colorectal cancer and an OR of 1.20 (95 % CI, 0.83–1.74), *P* for heterogeneity = 0.09; *I*^*2*^ = 65.7 % for cancer of upper aero-digestive tract (UADT) (Table 2). Subgroups analyses by different ethnicity showed a significant association between rs2470890 polymorphism and cancer for Mixed population OR = 1.44; 95 % CI 1.16–1.80; *I*^*2*^ = 0.0 %, P = 0.41, while not among Caucasians (OR = 1.07; 95 % CI 0.96–1.20; *I*^*2*^ = 0.0 %, *P* = 0.41) nor Asians (OR = 0.77; 95 % CI 0.37–1.64; *I*^*2*^ = 55.4 %, *P* = 0.13).

Results of the remaining three SNPs of *CYP1A2* are presented in the Fig. 3 and the Table 2. Absence of significant association with overall risk of cancer was reported. Only for rs2472304 we rendered an OR of 0.72 (95 % CI 0.52–0.99) *I*^*2*^ = 0.0 %, *P* = 0.61 for Caucasians, when doing a subgroup analyses on ethnicity. No evidence of significant heterogeneity was detected (data not shown).

When the meta-analyses were performed excluding small sample size studies for all examined SNPs, there were still no significant results obtained for the association between *CYP1A2* SNPs and cancer risk (Table 2).

## Discussion

The current meta-analysis included 71 studies with more than 47,000 cancer cases and 58,000 controls, detailing on all the *CYP1A2* gene polymorphisms and risk of cancer, shows no significant effect of investigated *CYP1A2* SNPs on cancer overall risk under various genetic models*.* Meta-analysis is a common tool for summarizing different studies to resolve the problem of small size statistical power and discrepancy in genetic association studies [85] and also it provides more reliable results than a single case-control study. To the best of our knowledge, this is the largest and most comprehensive meta-analysis on *CYP1A2* SNPs and cancer performed so far. Several previous meta-analyses have been reported on the association between *CYP1A2* gene polymorphisms and risk of cancer [86–95]. Deng et al. [87] reported no association between *CYP1A2* rs762551 polymorphism and lung cancer risk by including 1675 cases and 2393 controls. In the paper of Xue et al. [94], combined mutational homozygous and wild type homozygous genotype compared with mutational heterozygous genotype, had protective effect against gastric cancer by including 383 cases and 1229 controls. Wen-Xia Sun et al. [91] reported a significant protective effect of homozygous mutant of rs762551 *CYP1A2* SNP on bladder cancer in Caucasian population. Based on 19 studies, Wang et al. [93] found a borderline significantly increased risk of overall cancer among homozygous mutant of *CYP1A2* rs762551, mainly in Caucasians. The meta-analysis of 46 case-control studies by Tian et al. [92] suggested that the wild-type allele of *CYP1A2* rs762551 polymorphism might be associated with breast and ovarian cancer risk, especially among Caucasians. These inconclusive results could be explained by differences in study design, sample size, ethnicity, and cancer subtypes included.

The *CYP1A2* gene is a member of the CYP1 family and is involved in metabolism of carcinogens and estrogens. In particular, it plays an essential role in the metabolic activation of pro-carcinogens, such as polycyclic aromatic hydrocarbons (PAHs) and heterocyclic aromatic amines (HAA) [93]. Therefore, increased levels of this enzyme could explain the association with increased risk for cancer [16]. The wild genotype of CYP1A2*1 F represents a highly inducible genotype, and this high CYP1A2 activity may increase the hydroxylated forms as proximate carcinogens, from HCAs and aryl-amines [29].

In our meta-analyses, we showed that none of the investigated *CYP1A2* polymorphisms were significantly associated with overall risk of cancer at various sites. These results confirm the findings of a recent meta-analysis from Li Zhenzhen et al. [95] where was reported no significant associations with cancer risk in any genetic model (allele contrast, codominant, dominant, or recessive model) in terms of rs2069514 and rs3569413. For rs762551, they found that carriers of C-allele have an increased overall risk of developing cancer in allele genetic model (C-allele vs. A-allele) while not in other models. Their further subgroup analyses demonstrated that rs762551 polymorphism was associated with an increased risk of cancer in Caucasians under dominant model, while we investigated rs762551 under recessive model and did not obtain significant association. Moreover, their meta-analysis included only 37 case-control studies of rs762551 involving 16,825 cancer cases and 21,513 controls. Our meta-analysis may be the most comprehensive meta-analysis of the relationship between the *CYP1A2* rs762551 polymorphisms and the risk of cancer, to date.

When stratifying according to tumour site, our results showed a borderline not significant OR of 0.84 (95 % CI, 0.70–1.01) for bladder cancer for those homozygous mutant of rs762551 with total of 3430 cases and 3242 controls included (Table 2), thus confronting the previous evidence from Wen-Xia Sun et al. [91] that reported an OR = 0.79 (95 % CI 0.66–0.94) from 2415 cases and 2208 controls, and suggesting that on even bigger number of subjects investigated, this significance might disappear. Pavanello et al. [96] stressed that polymorphisms of rs762551 might be the crucial modulating factor along the continuum from the exposure to relevant environmental and occupational factors, in increased CYP1A2 activity of smokers measured by the urinary caffeine metabolic ratio.

We also found a significant decreased risk for bladder cancer for mutant carriers of rs762551 among the hospital-based population. Hospital-based studies have certain biases since those controls may have some benign diseases which can progress and also may not be representative of the general population. Using a population-based control would reduce the chance of bias in these studies.

In one recent meta-analysis by Zhi-Bin Bu et al. [86] on the association between *CYP1A2* rs762551, rs2069514, rs2069526, and rs2470890 polymorphisms and lung cancer risk, there was no evidence of significant association between lung cancer risk and *CYP1A2* rs2069514, s2470890, and rs2069526 polymorphisms. They found increased lung cancer risk for rs762551 polymorphism in Caucasians from 3 studies, while in our analysis there was no such connection on a bigger sample of studies [24, 26–28, 30–32].

Lastly, when stratifying our results for breast and colorectal cancer, we did not report any significant association between rs762551 and these cancers, thus confirming previous meta-analyses of Li-Xin Qiu et al. [90] on breast and Xiao-Feng He et al. [88] on colorectal cancer risk. Other meta-analysis by Jianbing Hu et al. [89] also suggested that *CYP1A2* rs762551 polymorphism was not a risk factor for colorectal cancer susceptibility, since no association was detected after all studies were pooled together nor in a subgroup analysis by ethnicity or source of controls, in all genetic models. The influence of the different *CYP1A2* SNPs might be camouflaged by the presence of some yet unidentified causal genes involved in many other types of cancer.

When stratifying the results according to ethnicity, the protective effect of rs2472304 in our study was restricted only to Caucasians, while for rs2470890, we noticed an increased risk among a mixed population. A possible explanation for these results could be that the same polymorphisms may play different roles in cancer susceptibility in different ethnic populations as well as different tumour positions, due to a difference in genetic backgrounds, the environment they live in, lifestyle and migrations, which all may have a critical role in cancer pathogenesis [97]. Also, some low penetrance genetic effects of single polymorphism could be determined by their interaction with other polymorphisms and/or a specific environmental exposure.

No other relevant results were reported for the remaining SNPs, however there were available only few studies regarding these associations, involving relatively small number of participants.

In interpreting the results, some limitations of our study should be considered. Firstly, only published studies were included, so there was space for publication bias, which in fact was confirmed by formal statistical tests. Secondly, the study size for most of the *CYP1A2* polymorphisms was limited to perform any meaningful subgroup analyses. Thirdly, it would have been valuable to stratify the results according to environmental effect modifiers, though this was not possible, as the original data sets were not available. Indeed, due to lack of access to original data used in included studies, our meta-analyses are based on the unadjusted data, so the effects might be confounded or modified by relevant covariates. Fourthly, beside breast cancer, there are no genome-wide association studies of the effects of *CYP1A2* polymorphisms on cancer risk. We were able to include only one breast cancer GWAS into our analyses, therefore our results might be affected by additional publication bias.

Despite these limitations, our meta-analyses also have some advantages. First, the statistical power of the analyses was noticeably increased as a huge number of cases and controls were pooled from different studies and has more statistical powerful than any single case-control study. Secondly, in our analyses, we included more studies than any previously published meta-analysis on the association between *CYP1A2* polymorphism and cancer risks and investigated 6 different *CYP1A2* SNPs.

## Conclusions

In conclusion, our meta-analysis suggests that investigated *CYP1A2* polymorphisms are not associated with cancer susceptibility under various genetic models. In order to reach a more definitive conclusion, there is a necessity for further gene-gene and gene-environment interaction studies to be conducted on different populations and larger sample size, for diverse *CYP1A2* SNPs.

## Abbreviations

95 % CI: 95 % confidence interval
CYP1A2: cytochrome P450 1A2
dbGaP: The database of Genotypes and Phenotypes
GAME-ON: Genetic Associations and Mechanisms in Oncology
GWAS: genome-wide association studies
GWAS DB: The genome wide association database
HAA: heterocyclic aromatic amines
HAs: heterocyclic amines
HuGE: the Human Genome Epidemiology Navigator
HWE: Hardy-Weinberg Equilibrium
mtmt: homozygous mutant genotype
NCBI: National Center for Biotechnology Information
NHGRI: National Human Genome Research Institute Catalog
ORs: Odds Ratios
PAHs: polycyclic aromatic hydrocarbons
PIs: principal investigators
SNPs: single nucleotide polymorphisms
VaDE: VarySysDB Disease Edition
wtmt: wild-type mutant-type heterozygous genotype
wtwt: wild-type homozygous genotype

## References

[1] Boffetta P, Boccia S, La Vecchia C. A Quick Guide to Cancer Epidemiology. Springer International Publishing; 2014. p. 11–4.

[2] Heller F (2013). Genetics/genomics and drug effects. Acta Clin Belg.

[3] Raunio H, Husgafvel-Pursiainen K, Anttila S, Hietanen E, Hirvonen A, Pelkonen O (1995). Diagnosis of polymorphisms in carcinogen-activating and inactivating enzymes and cancer susceptibility--a review. Gene.

[4] Altayli E, Gunes S, Yilmaz AF, Goktas S, Bek Y (2009). CYP1A2, CYP2D6, GSTM1, GSTP1, and GSTT1 gene polymorphisms in patients with bladder cancer in a Turkish population. Int Urol Nephrol.

[5] Sachse C, Bhambra U, Smith G, Lightfoot TJ, Barrett JH, Scollay J (2003). Polymorphisms in the cytochrome P450 CYP1A2 gene (CYP1A2) in colorectal cancer patients and controls: allele frequencies, linkage disequilibrium and influence on caffeine metabolism. Br J Clin Pharmacol.

[6] Nakajima M, Yokoi T, Mizutani M, Kinoshita M, Funayama M, Kamataki T (1999). Genetic polymorphism in the 5′-flanking region of human CYP1A2 gene: effect on the CYP1A2 inducibility in humans. J Biochem.

[7] Aldrich MC, Selvin S, Hansen HM, Barcellos LF, Wrensch MR, Sison JD (2009). CYP1A1/2 haplotypes and lung cancer and assessment of confounding by population stratification. Cancer Res.

[8] Li D, Jiao L, Li Y, Doll MA, Hein DW, Bondy ML (2006). Polymorphisms of cytochrome P4501A2 and N-acetyltransferase genes, smoking, and risk of pancreatic cancer. Carcinogenesis.

[9] Bae SY, Choi SK, Kim KR, Park CS, Lee SK, Roh HK (2006). Effects of genetic polymorphisms of MDR1, FMO3 and CYP1A2 on susceptibility to colorectal cancer in Koreans. Cancer Sci.

[10] Cleary SP, Cotterchio M, Shi E, Gallinger S, Harper P (2010). Cigarette smoking, genetic variants in carcinogen-metabolizing enzymes, and colorectal cancer risk. Am J Epidemiol.

[11] Cotterchio M, Boucher BA, Manno M, Gallinger S, Okey AB, Harper PA (2008). Red meat intake, doneness, polymorphisms in genes that encode carcinogen-metabolizing enzymes, and colorectal cancer risk. Cancer Epidemiol Biomarkers Prev.

[12] Dik VK, van Oijen MG, Uiterwaal C, van Gils C, van Duijnhoven FJ, Cauchi S (2013). Coffee consumption, genetic polymorphisms in CYP1A2 and NAT2, and colorectal cancer risk. Gastroenterology.

[13] Kiss I, Orsos Z, Gombos K, Bogner B, Csejtei A, Tibold A (2007). Association between allelic polymorphisms of metabolizing enzymes (CYP 1A1, CYP 1A2, CYP 2E1, mEH) and occurrence of colorectal cancer in Hungary. Anticancer Res.

[14] Kobayashi M, Otani T, Iwasaki M, Natsukawa S, Shaura K, Koizumi Y (2009). Association between dietary heterocyclic amine levels, genetic polymorphisms of NAT2, CYP1A1, and CYP1A2 and risk of colorectal cancer: a hospital-based case-control study in Japan. Scand J Gastroenterol.

[15] Kury S, Buecher B, Robiou-du-Pont S, Scoul C, Sebille V, Colman H (2007). Combinations of cytochrome P450 gene polymorphisms enhancing the risk for sporadic colorectal cancer related to red meat consumption. Cancer Epidemiol Biomarkers Prev.

[16] Landi S, Gemignani F, Moreno V, Gioia-Patricola L, Chabrier A, Guino E (2005). A comprehensive analysis of phase I and phase II metabolism gene polymorphisms and risk of colorectal cancer. Pharmacogenet Genomics.

[17] Rudolph A, Sainz J, Hein R, Hoffmeister M, Frank B, Forsti A (2011). Modification of menopausal hormone therapy-associated colorectal cancer risk by polymorphisms in sex steroid signaling, metabolism and transport related genes. Endocr Relat Cancer.

[18] Sachse C, Smith G, Wilkie MJ, Barrett JH, Waxman R, Sullivan F (2002). A pharmacogenetic study to investigate the role of dietary carcinogens in the etiology of colorectal cancer. Carcinogenesis.

[19] Saebo M, Skjelbred CF, Brekke Li K, Bowitz Lothe IM, Hagen PC, Johnsen E (2008). CYP1A2 164 A-->C polymorphism, cigarette smoking, consumption of well-done red meat and risk of developing colorectal adenomas and carcinomas. Anticancer Res.

[20] Sainz J, Rudolph A, Hein R, Hoffmeister M, Buch S, von Schonfels W (2011). Association of genetic polymorphisms in ESR2, HSD17B1, ABCB1, and SHBG genes with colorectal cancer risk. Endocr Relat Cancer.

[21] Wang J, Joshi AD, Corral R, Siegmund KD, Marchand LL, Martinez ME (2012). Carcinogen metabolism genes, red meat and poultry intake, and colorectal cancer risk. Int J Cancer.

[22] Yeh CC, Sung FC, Tang R, Chang-Chieh CR, Hsieh LL (2009). Polymorphisms of cytochrome P450 1A2 and N-acetyltransferase genes, meat consumption, and risk of colorectal cancer. Dis Colon Rectum.

[23] Yoshida K, Osawa K, Kasahara M, Miyaishi A, Nakanishi K, Hayamizu S (2007). Association of CYP1A1, CYP1A2, GSTM1 and NAT2 gene polymorphisms with colorectal cancer and smoking. Asian Pac J Cancer Prev.

[24] B’Chir F, Pavanello S, Knani J, Boughattas S, Arnaud MJ, Saguem S (2009). CYP1A2 genetic polymorphisms and adenocarcinoma lung cancer risk in the Tunisian population. Life Sci.

[25] Chiou HL, Wu MF, Chien WP, Cheng YW, Wong RH, Chen CY (2005). NAT2 fast acetylator genotype is associated with an increased risk of lung cancer among never-smoking women in Taiwan. Cancer Lett.

[26] Gemignani F, Landi S, Szeszenia-Dabrowska N, Zaridze D, Lissowska J, Rudnai P (2007). Development of lung cancer before the age of 50: the role of xenobiotic metabolizing genes. Carcinogenesis.

[27] Gervasini G, Ghotbi R, Aklillu E, San Jose C, Cabanillas A, Kishikawa J (2013). Haplotypes in the 5′-untranslated region of the CYP1A2 gene are inversely associated with lung cancer risk but do not correlate with caffeine metabolism. Environ Mol Mutagen.

[28] Mikhalenko AP, Krupnova EV, Chakova NN, Chebotareva NV, Demidchik YE (2014). Assessment of the relationship between combinations of polymorphic variants of xenobiotic-metabolizing enzyme genes and predisposition to lung cancer. Cytol Genet.

[29] Osawa Y, Osawa KK, Miyaishi A, Higuchi M, Tsutou A, Matsumura S (2007). NAT2 and CYP1A2 polymorphisms and lung cancer risk in relation to smoking status. Asian Pac J Cancer Prev.

[30] Pavanello S, Fedeli U, Mastrangelo G, Rota F, Overvad K, Raaschou-Nielsen O (2012). Role of CYP1A2 polymorphisms on lung cancer risk in a prospective study. Cancer Genet.

[31] Singh AP, Pant MC, Ruwali M, Shah PP, Prasad R, Mathur N (2010). Polymorphism in cytochrome P450 1A2 and their interaction with risk factors in determining risk of squamous cell lung carcinoma in men. Cancer Biomark.

[32] Zienolddiny S, Campa D, Lind H, Ryberg D, Skaug V, Stangeland LB (2008). A comprehensive analysis of phase I and phase II metabolism gene polymorphisms and risk of non-small cell lung cancer in smokers. Carcinogenesis.

[33] Anderson LN, Cotterchio M, Mirea L, Ozcelik H, Kreiger N (2012). Passive cigarette smoke exposure during various periods of life, genetic variants, and breast cancer risk among never smokers. Am J Epidemiol.

[34] Ayari I, Fedeli U, Saguem S, Hidar S, Khlifi S, Pavanello S (2013). Role of CYP1A2 polymorphisms in breast cancer risk in women. Mol Med Rep.

[35] Gulyaeva LF, Mikhailova ON, PustyInyak VO, Kim IV, Gerasimov AV, Krasilnikov SE (2008). Comparative analysis of SNP in estrogen-metabolizing enzymes for ovarian, endometrial, and breast cancers in Novosibirsk, Russia. Adv Exp Med Biol.

[36] Hopper JL, Dite GS, Jenkins MA, Southey MC, Hocking JS, Giles GG (2003). Familial risks, early-onset breast cancer, and BRCA1 and BRCA2 germline mutations. J Natl Cancer Inst.

[37] Khvostova EP, Pustylnyak VO, Gulyaeva LF (2012). Genetic polymorphism of estrogen metabolizing enzymes in Siberian women with breast cancer. Genet Test Mol Biomarkers.

[38] Kotsopoulos J, Ghadirian P, El-Sohemy A, Lynch HT, Snyder C, Daly M (2007). The CYP1A2 genotype modifies the association between coffee consumption and breast cancer risk among BRCA1 mutation carriers. Cancer Epidemiol Biomarkers Prev.

[39] Le Marchand L, Donlon T, Kolonel LN, Henderson BE, Wilkens LR (2005). Estrogen metabolism-related genes and breast cancer risk: the multiethnic cohort study. Cancer Epidemiol Biomarkers Prev.

[40] Lee HJ, Wu K, Cox DG, Hunter D, Hankinson SE, Willett WC (2013). Polymorphisms in xenobiotic metabolizing genes, intakes of heterocyclic amines and red meat, and postmenopausal breast cancer. Nutr Cancer.

[41] Long JR, Egan KM, Dunning L, Shu XO, Cai Q, Cai H (2006). Population-based case-control study of AhR (aryl hydrocarbon receptor) and CYP1A2 polymorphisms and breast cancer risk. Pharmacogenet Genomics.

[42] Lowcock EC, Cotterchio M, Anderson LN, Boucher BA, El-Sohemy A (2013). High coffee intake, but not caffeine, is associated with reduced estrogen receptor negative and postmenopausal breast cancer risk with no effect modification by CYP1A2 genotype. Nutr Cancer.

[43] Marie-Genica C (2010). Genetic polymorphisms in phase I and phase II enzymes and breast cancer risk associated with menopausal hormone therapy in postmenopausal women. Breast Cancer Res Treat.

[44] Sangrajrang S, Sato Y, Sakamoto H, Ohnami S, Laird NM, Khuhaprema T (2009). Genetic polymorphisms of estrogen metabolizing enzyme and breast cancer risk in Thai women. Int J Cancer.

[45] Shimada N, Iwasaki M, Kasuga Y, Yokoyama S, Onuma H, Nishimura H (2009). Genetic polymorphisms in estrogen metabolism and breast cancer risk in case-control studies in Japanese, Japanese Brazilians and non-Japanese Brazilians. J Hum Genet.

[46] Takata Y, Maskarinec G, Le Marchand L (2007). Breast density and polymorphisms in genes coding for CYP1A2 and COMT: the Multiethnic Cohort. BMC Cancer.

[47] Cui X, Lu X, Hiura M, Omori H, Miyazaki W, Katoh T (2013). Association of genotypes of carcinogen-metabolizing enzymes and smoking status with bladder cancer in a Japanese population. Environ Health Prev Med.

[48] Figueroa JD, Malats N, Garcia-Closas M, Real FX, Silverman D, Kogevinas M (2008). Bladder cancer risk and genetic variation in AKR1C3 and other metabolizing genes. Carcinogenesis.

[49] Guey LT, Garcia-Closas M, Murta-Nascimento C, Lloreta J, Palencia L, Kogevinas M (2010). Genetic susceptibility to distinct bladder cancer subphenotypes. Eur Urol.

[50] Pavanello S, Mastrangelo G, Placidi D, Campagna M, Pulliero A, Carta A (2010). CYP1A2 polymorphisms, occupational and environmental exposures and risk of bladder cancer. Eur J Epidemiol.

[51] Tsukino H, Kuroda Y, Nakao H, Imai H, Inatomi H, Osada Y (2004). Cytochrome P450 (CYP) 1A2, sulfotransferase (SULT) 1A1, and N-acetyltransferase (NAT) 2 polymorphisms and susceptibility to urothelial cancer. J Cancer Res Clin Oncol.

[52] Villanueva CM, Silverman DT, Murta-Nascimento C, Malats N, Garcia-Closas M, Castro F (2009). Coffee consumption, genetic susceptibility and bladder cancer risk. Cancer Causes Control.

[53] National Center for Biotechnology Information. dbSNP Home Page. [cited 2015 02/15]. Available from: http://www.ncbi.nlm.nih.gov/SNP/index.html. Accessed 15 Feb 2015.

[54] Attia J, Thakkinstian A, D’Este C (2003). Meta-analyses of molecular association studies: methodologic lessons for genetic epidemiology. J Clin Epidemiol.

[55] DerSimonian R, Laird N (1986). Meta-analysis in clinical trials. Control Clin Trials.

[56] Deeks J, Altman D, Bradburn M, Egger M, Davey Smith G, Altman D (2001). Statistical methods for examining heterogeneity and combining results from several studies in meta-analysis. Systematic reviews in health care: meta-analysis in context.

[57] Higgins JP, Thompson SG, Deeks JJ, Altman DG (2003). Measuring inconsistency in meta-analyses. BMJ.

[58] Egger M, Davey Smith G, Schneider M, Minder C (1997). Bias in meta-analysis detected by a simple, graphical test. BMJ.

[59] Ioannidis JP, Trikalinos TA (2007). The appropriateness of asymmetry tests for publication bias in meta-analyses: a large survey. CMAJ.

[60] Galbraith RF (1988). A note on graphical presentation of estimated odds ratios from several clinical trials. Stat Med.

[61] Agudo A, Sala N, Pera G, Capella G, Berenguer A, Garcia N (2006). Polymorphisms in metabolic genes related to tobacco smoke and the risk of gastric cancer in the European prospective investigation into cancer and nutrition. Cancer Epidemiol Biomarkers Prev.

[62] Ashton KA, Proietto A, Otton G, Symonds I, McEvoy M, Attia J (2010). Polymorphisms in genes of the steroid hormone biosynthesis and metabolism pathways and endometrial cancer risk. Cancer Epidemiol.

[63] Barbieri RB, Bufalo NE, Cunha LL, Assumpcao LV, Maciel RM, Cerutti JM (2013). Genes of detoxification are important modulators of hereditary medullary thyroid carcinoma risk. Clin Endocrinol.

[64] Canova C, Hashibe M, Simonato L, Nelis M, Metspalu A, Lagiou P (2009). Genetic associations of 115 polymorphisms with cancers of the upper aerodigestive tract across 10 European countries: the ARCAGE project. Cancer Res.

[65] Canova C, Richiardi L, Merletti F, Pentenero M, Gervasio C, Tanturri G (2010). Alcohol, tobacco and genetic susceptibility in relation to cancers of the upper aerodigestive tract in northern Italy. Tumori.

[66] Chen X, Wang H, Xie W, Liang R, Wei Z, Zhi L (2006). Association of CYP1A2 genetic polymorphisms with hepatocellular carcinoma susceptibility: a case-control study in a high-risk region of China. Pharmacogenet Genomics.

[67] De Roos AJ, Gold LS, Wang S, Hartge P, Cerhan JR, Cozen W (2006). Metabolic gene variants and risk of non-Hodgkin’s lymphoma. Cancer Epidemiol Biomarkers Prev.

[68] Doherty JA, Weiss NS, Freeman RJ, Dightman DA, Thornton PJ, Houck JR (2005). Genetic factors in catechol estrogen metabolism in relation to the risk of endometrial cancer. Cancer Epidemiol Biomarkers Prev.

[69] Eom SY, Yim DH, Zhang Y, Yun JK, Moon SI, Yun HY (2013). Dietary aflatoxin B1 intake, genetic polymorphisms of CYP1A2, CYP2E1, EPHX1, GSTM1, and GSTT1, and gastric cancer risk in Korean. Cancer Causes Control.

[70] Ferlin A, Ganz F, Pengo M, Selice R, Frigo AC, Foresta C (2010). Association of testicular germ cell tumor with polymorphisms in estrogen receptor and steroid metabolism genes. Endocr Relat Cancer.

[71] Gemignani F, Neri M, Bottari F, Barale R, Canessa PA, Canzian F (2009). Risk of malignant pleural mesothelioma and polymorphisms in genes involved in the genome stability and xenobiotics metabolism. Mutat Res.

[72] Ghoshal U, Tripathi S, Kumar S, Mittal B, Chourasia D, Kumari N (2014). Genetic polymorphism of cytochrome P450 (CYP) 1A1, CYP1A2, and CYP2E1 genes modulate susceptibility to gastric cancer in patients with Helicobacter pylori infection. Gastric Cancer.

[73] Goodman MT, McDuffie K, Kolonel LN, Terada K, Donlon TA, Wilkens LR (2001). Case-control study of ovarian cancer and polymorphisms in genes involved in catecholestrogen formation and metabolism. Cancer Epidemiol Biomarkers Prev.

[74] Goodman MT, Tung KH, McDuffie K, Wilkens LR, Donlon TA (2003). Association of caffeine intake and CYP1A2 genotype with ovarian cancer. Nutr Cancer.

[75] Hirata H, Hinoda Y, Okayama N, Suehiro Y, Kawamoto K, Kikuno N (2008). CYP1A1, SULT1A1, and SULT1E1 polymorphisms are risk factors for endometrial cancer susceptibility. Cancer.

[76] Imaizumi T, Higaki Y, Hara M, Sakamoto T, Horita M, Mizuta T (2009). Interaction between cytochrome P450 1A2 genetic polymorphism and cigarette smoking on the risk of hepatocellular carcinoma in a Japanese population. Carcinogenesis.

[77] Jang JH, Cotterchio M, Borgida A, Gallinger S, Cleary SP (2012). Genetic variants in carcinogen-metabolizing enzymes, cigarette smoking and pancreatic cancer risk. Carcinogenesis.

[78] Kobayashi M, Otani T, Iwasaki M, Natsukawa S, Shaura K, Koizumi Y (2009). Association between dietary heterocyclic amine levels, genetic polymorphisms of NAT2, CYP1A1, and CYP1A2 and risk of stomach cancer: a hospital-based case-control study in Japan. Gastric Cancer.

[79] Mochizuki J, Murakami S, Sanjo A, Takagi I, Akizuki S, Ohnishi A (2005). Genetic polymorphisms of cytochrome P450 in patients with hepatitis C virus-associated hepatocellular carcinoma. J Gastroenterol Hepatol.

[80] Olivieri EH, da Silva SD, Mendonca FF, Urata YN, Vidal DO, Faria Mde A (2009). CYP1A2*1C, CYP2E1*5B, and GSTM1 polymorphisms are predictors of risk and poor outcome in head and neck squamous cell carcinoma patients. Oral Oncol.

[81] Prawan A, Kukongviriyapan V, Tassaneeyakul W, Pairojkul C, Bhudhisawasdi V (2005). Association between genetic polymorphisms of CYP1A2, arylamine N-acetyltransferase 1 and 2 and susceptibility to cholangiocarcinoma. Eur J Cancer Prev.

[82] Rebbeck TR, Troxel AB, Wang Y, Walker AH, Panossian S, Gallagher S (2006). Estrogen sulfation genes, hormone replacement therapy, and endometrial cancer risk. J Natl Cancer Inst.

[83] Shahabi A, Corral R, Catsburg C, Joshi AD, Kim A, Lewinger JP (2014). Tobacco smoking, polymorphisms in carcinogen metabolism enzyme genes, and risk of localized and advanced prostate cancer: results from the California Collaborative Prostate Cancer Study. Cancer Med.

[84] Suzuki H, Morris JS, Li Y, Doll MA, Hein DW, Liu J (2008). Interaction of the cytochrome P4501A2, SULT1A1 and NAT gene polymorphisms with smoking and dietary mutagen intake in modification of the risk of pancreatic cancer. Carcinogenesis.

[85] Munafo MR, Flint J (2004). Meta-analysis of genetic association studies. Trends Genet.

[86] Bu ZB, Ye M, Cheng Y, Wu WZ (2014). Four polymorphisms in the cytochrome P450 1A2 (CYP1A2) gene and lung cancer risk: a meta-analysis. Asian Pac J Cancer Prev.

[87] Deng SQ, Zeng XT, Wang Y, Ke Q, Xu QL (2013). Meta-analysis of the CYP1A2 -163C>A polymorphism and lung cancer risk. Asian Pac J Cancer Prev.

[88] He XF, Wei J, Liu ZZ, Xie JJ, Wang W, Du YP (2014). Association between CYP1A2 and CYP1B1 polymorphisms and colorectal cancer risk: a meta-analysis. PLoS One.

[89] Hu J, Liu C, Yin Q, Ying M, Li J, Li L (2014). Association between the CYP1A2-164 A/C polymorphism and colorectal cancer susceptibility: a meta-analysis. Mol Genet Genomics.

[90] Qiu LX, Yao L, Mao C, Yu KD, Zhan P, Chen B (2010). Lack of association of CYP1A2-164 A/C polymorphism with breast cancer susceptibility: a meta-analysis involving 17,600 subjects. Breast Cancer Res Treat.

[91] Sun WX, Chen YH, Liu ZZ, Xie JJ, Wang W, Du YP (2015). Association between the CYP1A2 polymorphisms and risk of cancer: a meta-analysis. Mol Genet Genomics.

[92] Tian Z, Li YL, Zhao L, Zhang CL (2013). Role of CYP1A2 1F polymorphism in cancer risk: evidence from a meta-analysis of 46 case-control studies. Gene.

[93] Wang H, Zhang Z, Han S, Lu Y, Feng F, Yuan J (2012). CYP1A2 rs762551 polymorphism contributes to cancer susceptibility: a meta-analysis from 19 case-control studies. BMC Cancer.

[94] Xue H, Lu Y, Xue Z, Lin B, Chen J, Tang F (2014). The effect of CYP1A1 and CYP1A2 polymorphisms on gastric cancer risk among different ethnicities: a systematic review and meta-analysis. Tumour Biol.

[95] Zhenzhen L, Xianghua L, Ning S, Zhan G, Chuanchuan R, Jie L (2013). Current evidence on the relationship between three polymorphisms in the CYP1A2 gene and the risk of cancer. Eur J Cancer Prev.

[96] Pavanello S, Pulliero A, Lupi S, Gregorio P, Clonfero E (2005). Influence of the genetic polymorphism in the 5′-noncoding region of the CYP1A2 gene on CYP1A2 phenotype and urinary mutagenicity in smokers. Mutat Res.

[97] Hirschhorn JN, Lohmueller K, Byrne E, Hirschhorn K (2002). A comprehensive review of genetic association studies. Genet Med.

